# Capsules from Pathogenic and Non-Pathogenic *Cryptococcus* spp. Manifest Significant Differences in Structure and Ability to Protect against Phagocytic Cells

**DOI:** 10.1371/journal.pone.0029561

**Published:** 2012-01-12

**Authors:** Glauber de S. Araujo, Fernanda L. Fonseca, Bruno Pontes, Andre Torres, Radames J. B. Cordero, Rosely M. Zancopé-Oliveira, Arturo Casadevall, Nathan B. Viana, Leonardo Nimrichter, Marcio L. Rodrigues, Eloi S. Garcia, Wanderley de Souza, Susana Frases

**Affiliations:** 1 Laboratório de Biotecnologia, Instituto Nacional de Metrologia, Normalização e Qualidade Industrial, Rio de Janeiro, Brazil; 2 Laboratório de Estudos integrados em Bioquímica Microbiana, Instituto de Microbiologia Professor Paulo de Góes, Universidade Federal do Rio de Janeiro, Rio de Janeiro, Brazil; 3 LPO-COPEA, Instituto de Ciências Biomédicas, Universidade Federal do Rio de Janeiro, Brazil; 4 Instituto de Pesquisa Clínica Evandro Chagas, The Oswaldo Cruz Foundation, Rio de Janeiro, Brazil; 5 Department of Microbiology and Immunology. Albert Einstein College of Medicine, Bronx, New York, United States of America; 6 Instituto de Física, Universidade Federal do Rio de Janeiro, Caixa, Brazil; 7 Instituto Oswaldo Cruz, Rio de Janeiro, Brazil; 8 Instituto Nacional de Ciência e Tecnologia em Biologia Estrutural e Bioimagens, Universidade Federal do Rio de Janeiro, Rio de Janeiro, Brazil; 9 Laboratório de Ultraestrutura Celular Hertha Meyer, Instituto de Biofísica Carlos Chagas Filho, Universidade Federal do Rio de Janeiro, Rio de Janeiro, Brazil; University of Minnesota, United States of America

## Abstract

Capsule production is common among bacterial species, but relatively rare in eukaryotic microorganisms. Members of the fungal *Cryptococcus* genus are known to produce capsules, which are major determinants of virulence in the highly pathogenic species *Cryptococcus neoformans* and *Cryptococcus gattii*. Although the lack of virulence of many species of the *Cryptococcus* genus can be explained solely by the lack of mammalian thermotolerance, it is uncertain whether the capsules from these organisms are comparable to those of the pathogenic cryptococci. In this study, we compared the characteristic of the capsule from the non-pathogenic environmental yeast *Cryptococcus liquefaciens* with that of *C. neoformans*. Microscopic observations revealed that *C. liquefaciens* has a capsule visible in India ink preparations that was also efficiently labeled by three antibodies generated to specific *C. neoformans* capsular antigens. Capsular polysaccharides of *C. liquefaciens* were incorporated onto the cell surface of acapsular *C. neoformans* mutant cells. Polysaccharide composition determinations in combination with confocal microscopy revealed that *C. liquefaciens* capsule consisted of mannose, xylose, glucose, glucuronic acid, galactose and N-acetylglucosamine. Physical chemical analysis of the *C. liquefaciens* polysaccharides in comparison with *C. neoformans* samples revealed significant differences in viscosity, elastic properties and macromolecular structure parameters of polysaccharide solutions such as rigidity, effective diameter, zeta potential and molecular mass, which nevertheless appeared to be characteristics of linear polysaccharides that also comprise capsular polysaccharide of *C. neoformans*. The environmental yeast, however, showed enhanced susceptibility to the antimicrobial activity of the environmental phagocytes, suggesting that the *C. liquefaciens* capsular components are insufficient in protecting yeast cells against killing by amoeba. These results suggest that capsular structures in pathogenic *Cryptococcus* species and environmental species share similar features, but also manifest significant difference that could influence their potential to virulence.

## Introduction

Species belonging to the *Cryptococcus* genus are widely distributed in nature. *Cryptococcus* spp. can be isolated from various environmental sources such as air, soil, bird excreta, water, animals and decomposing wood [1]. Within the genus, only a few species are considered medically important and these appear to have different characteristics that confer virulence [2]. The species mainly responsible for disease in man and animals are *Cryptococcus neoformans* and *Cryptococcus gattii* [3]. However, in recent years there has been an increased incidence of infections caused by other species, such as *Cryptococcus laurentii* and *Cryptococcus albidus*, which together are responsible for 80% of non-*neoformans* and non-*gattii* cryptococcosis cases [4]–[7]. Understanding the characteristics that separate the pathogenic and non-pathogenic cryptococcal species is important for ascertaining the virulence potential of various members of this genus. Such insights may be important is anticipating the emergence of new virulent fungi as it has been hypothesized that climate warming promotes increased thermotolerance for fungal species [8] and that mammalian temperatures are optimally fit for resisting fungal pathogens [9].

*C. neoformans* and *C. gattii* possess a variety of well characterized virulence factors, such as polysaccharide (PS) capsule formation, melanin production and growth at 37°C. However, capsules are not limited to these pathogenic species as they are common in the Cryptococcus genus. Until now, little work has been done to understand whether the capsular structures of pathogenic and non-pathogenic cryptococcal species are comparable in function and in their virulence potential. For *C. neoformans* and *C. gattii*, the capsule is known to have protean functions in virulence ranging from protecting the fungal cell against host antimicrobial mechanisms to interfering with immune responses [10]–[14]. There is no data assessing whether capsules from other members of the *Cryptococcus* genus posses similar properties. This question is important for efforts to understand the origin of virulence and the characteristics that separate pathogenic and non-pathogenic members of this genus.

Capsular PS complexity in *C. neoformans* has been extensively study [15]–[18]. GXM is composed of combinations of seven structural motifs that differ in the degree of acetylation in mannose residues [19]–[22]. Generating capsules that vary in the ratio of these residues provides an enormous combinatorial diversity such that an infinitively vast number of structural possibilities are possible [22]. In recent years our group has applied different physical-chemical techniques to study biophysical parameters of cryptococcal PS, including static and dynamic light scattering, viscoelastic properties analyses and high-resolution microscopy [23]–[27]. These approaches have produced new insights into the relationship between capsule structure and function. To better understand what is unique and what is shared among cryptococcal capsules we analyzed the capsule of a non-pathogenic, environmental member of the cryptococcal genus, *C. liquifaciens* and compared this species PS with that of *C. neoformans*. *C. liquifaciens* is not pathogenic for mammals and its inability to cause disease has been attributed to a lack of thermolerance at mammalian temperatures. However, *C. liquifaciens* expresses a pronounced capsule, and thus provides a reasonable reference point for an initial comparative study. In this study, we have employed a combination of microscopy, physical chemistry and serology to characterize the capsule of *C. liquefaciens*. Our goal is to identify differences that could provide insight into the requirements for capsules to contribute to pathogenicity. Our results reveal that *C. neoformans* and *C. liquefaciens* capsules share many characteristics but also differed in key aspects, which may be associated with the variable ability of each species to cause disease in an animal host and to survive in the environment.

## Results

### Isolation and identification of *C. liquefaciens* from *Achatina fulica*

**Figure 1:**
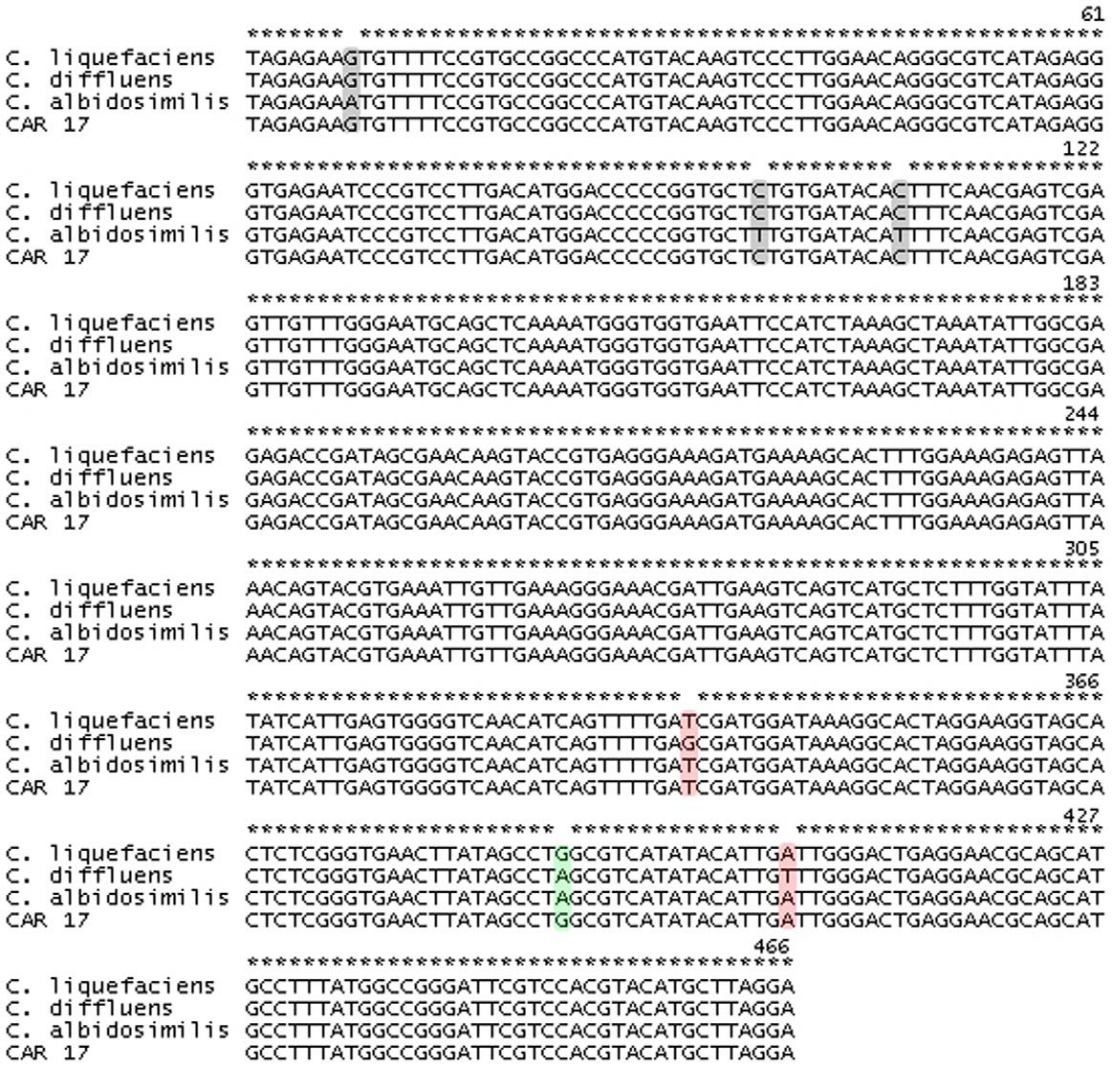
Alignment of ribosomal region D1/D2, in 28 S rDNA, between *C. liquefaciens* strain Car17 and related species.

Fungal cells isolated from digestive tract of snails were subcloned to purity on Sabouraud agar and analyzed by biochemical and molecular biology identification methods. One isolate, *car17*, was selected for in depth analysis. Molecular identification of the *car17* strain was done using two ribosomal DNA targets. ITS-5.8 S rDNA showed 99% maximum identity and 97% query coverage between *Car17* sequence and *C. liquefaciens, Cryptococcus albidosimilis* and *Cryptococcus diffluens* sequences. To discriminate between these three closely related species, we used a second ribosomal target (D1–D2 region on 28 S rDNA), which is used to analyze highly related species. Alignment of *car17* sequence of this region showed 100% maximum identity and 100% query coverage with *C. liquefaciens*. Hence, the subtle differences in nucleotide composition between these highly related species allowed us to identify car17 as a *C. liquefaciens* (Figure 1).

**Figure 2:**
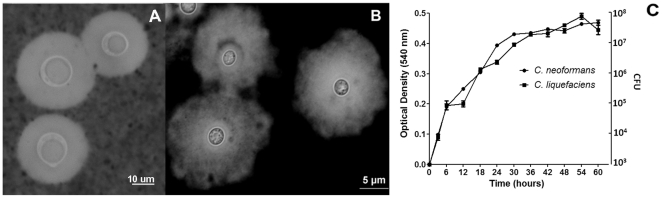
Microscopic visualization of *C. liquefaciens and C. neoformans capsules and growth profile*. Panel A) India ink stain of *C. neoformans* cells. Panel B) Counterstaining of *C. liquefaciens* with India ink. Panel C) Growth curves of *C. neoformans* and *C. liquefaciens* at 30°C in minimal media.

On Sabouraud dextrose agar, colonies of *C. liquifaciens car17* strain at 30°C were cream colored, smooth-mucoid and yeast-like in appearance. Microscopic examination revealed a globose to ovoid morphology with cell body diameters of 3.5±2.2 µm (data not shown). Yeast cells were cultivated in minimal media at 30°C, washed with PBS and suspended in India ink for microscopic evaluation. Both *C. neoformans* and *C. liquefaciens* produced large capsules (Figure 2). *C. liquefaciens* cells expressed very large capsules, which manifested different zones of density (Figure 2B). Regions of stronger staining were closer to the cell wall, while capsular areas with higher degrees of permeability to India ink were distributed into the periphery of the capsule. In contrast to *C. neoformans* and *C. gattii* [27]–[29], the *C. liquefaciens* capsule showed poorly defined borders. The capsule sizes were similar between the two species, with *C. neoformans* having an average radius of 5.6±1.3 µm and *C. liquefaciens* 5.1±1.2 µm (P value>0.05).

Physiological tests of *car17* strain revealed a lack of tube germ formation at 30°C, no urea hydrolysis, and there was no growth in cycloheximide medium and at either 4 or 37°C. No pigmentation was observed in *C. liquefaciens* cells after incubation with L-dopa for 20 days, suggesting that unlike *C. neoformans* this specie made no melanin. However, the growth dynamics of *car17* in minimal media at 30°C were nearly indistinguishable from those of *C. neoformans* H99 strain (Figure 1C).

### Compositional analysis of exo- and capsular-PS from *C. liquefaciens*

The existence of a capsular network in *C. liquefaciens* suggested that methods used for the isolation of capsular components in *C. neoformans* might be applicable to study the capsule of this environmental yeast. Therefore, *C. liquefaciens* and *C. neoformans* yeast cell cultures in medium were subjected to supernatant filtration to isolate exo-PS and DMSO to extract capsular-PS for subsequent monosaccharide determinations. As described for *C. neoformans*, ultrafiltration of *C. liquefaciens* supernatants resulted in the formation of an easily collectable polysaccharide gel on the filter membrane.Carbohydrate determinations for three different extractions according to the method of Dubois revealed that concentration of exo-PS from cultures of *C. liquefaciens* and *C. neoformans* at similar cell densities was 8.5±1.3 and 9.1±0.9 mg/ml, respectively (P value>0.05). DMSO extracts from whole cells revealed similar PS concentrations (5.4±1.3 mg/ml for *C. liquefaciens* and 4.9±0.6 mg/ml for *C. neoformans*; P value>0.05). These measurements indicated that both species produced approximately the same amount of both PS forms.

**Figure 3:**
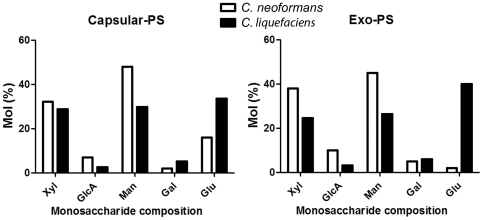
Monosaccharide composition of capsular and exo-PS of *C. liquefaciens* and *C. neoformans*. Xyl (Xylose), GlcA (Glucuronic acid), Man (Mannose), Gal (Galactose), Glu (Glucose) and GlcNAc (N-acetylglucosamine).

As expected, the *C. neoformans* DMSO extract (capsular-PS) revealed the presence of mannose, xylose and glucuronic acid, the components of criptococal polysaccharide (Figure 3 Table 1). Glucose was also detected in this fraction, which has been previously described in *C. neoformans* capsular-PS. *C. liquefaciens* PS fractions had more glucose compared to *C. neoformans* (33.5% vs 16.0%,). In contrast, *C. liquefaciens* fractions had lower concentrations relative to *C. neoformans* of glucuronic acid (2.6% vs 7.0%) and galactose (5.2% vs 2.0%). Interestingly, *C. liquefaciens* also had trace amounts of N-acetylglucosamine (0.5%). Similarly, in comparing exo-PS fractions from the two species, *C. neoformans* had increased amounts of xylose (24.6% vs 38.0%,), glucuronic acid (3.2% vs 10.2%), and mannose (26.4% vs 45.1%,) (Figure 3, Table 1). *C. liquefaciens* exo-PS fractions contained higher amounts of galactose and glucose (5.9% vs 39.9%,). Notably, N-acetylglucosamine was absent in the exo-PS fraction from *C. liquefaciens*.

**Table 1:** Monosaccharide composition for C. liquefaciens and C. neoformans PS.

	Capsular-PS		Exo-PS	
	*C. neoformans*	*C. liquefaciens*	*C. neoformans*	*C. liquefaciens*
Xylose	32.3	28.8	38.0	24.6
Glucuronic acid	7.0	2.6	10.2	3.2
Mannose	48.0	29.8	45.1	26.4
Galactose	2.0	5.2	5.0	5.9
Glucose	16.0	33.5	2.0	39.9
N-acetylglucosamine	NP	0.5	NP	NP

Values are percentage of mole. NP not presence.

### Physical chemical and hydrodynamic properties of exo- and capsular-PS

**Figure 4:**
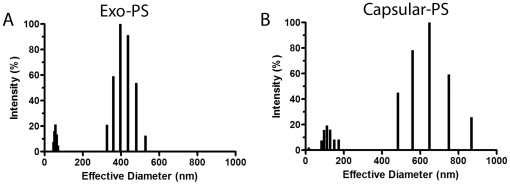
Physical properties of capsular and exo-PS from *C. liquefaciens* obtained by DLS. Panel A and B show size distribution of PS fibers capsular and exo-PS samples, respectively.

For the systematic analysis of the properties of the *C. liquefaciens* PS, a number of physical chemical parameters were evaluated. Average molecular weight and radius of gyration determinations were performed by static light scattering analysis of exo- and capsular-PS samples. Analysis of Exo-PS revealed a M_w_ of (1.04±0.69)×10^6^ g/mol and R_g_ of 222±84 nm. Capsular-PS showed an M_W_ of (1.05±0.29)×10^8^ g/mol and R_g_ of 191±32 nm. Hydrodynamic properties of *C. liquefaciens* PS were calculated from dynamic light scattering measurements. Exo-PS had a hydrodynamic radius (R_h_) of 145.9 nm and a diffusion coefficient of 1.68×10^−8^ cm^2^/s. Capsular-PS presented an R_h_ of 186.39 nm and diffusion coefficient of 1.32×10^−8^ cm^2^/s. Size distribution of PS fibers showed significant differences between exo- and capsular-PS (Figure 4A and B). By combining results from SLS and DLS we calculated the shape factor (ρ) of PS fractions from *C. liquefaciens*. The ρ of a polysaccharide is the ratio of R_g_ to R_h_ and is a measure of the degree of branching of a molecule. *C. liquefaciens* capsular-PS showed a ρ of 1.03 and exo-PS a value of 1.521. PS fractions obtained under the same conditions from *C. neoformans* demonstrate values of 0.42 (exo-PS) and 0.28 (capsular-PS).

**Figure 5:**
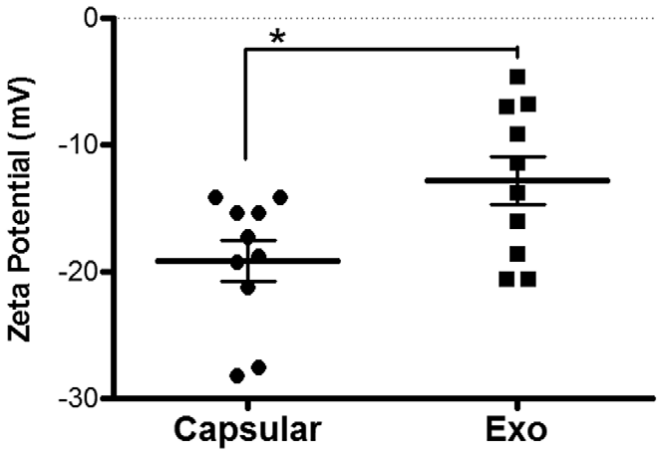
Zeta Potential of exo- and capsular-PS from *C. liquefaciens*. The charges of the different *C. liquefaciens* PS forms were significantly different (P Value<0.01 calculated by *Student's T test (two-tailed)*).

Zeta potentials of PS fractions were measured for *C. liquefaciens* and compared with *C. neoformans*. *C. liquefaciens* exo- and capsular-PS showed zeta potential averages of −12.87±5.95 mV and −19.16±5.17 mV, respectively (Figure 5). Zeta potential, mobility and frequency shift were significantly different in both fractions (P value = 0.01). For *C. neoformans*, the average zeta potentials for both exo- and capsular PS were negative, being less than −30 mV under the same conditions [25], [30]. The smaller content of glucuronic acid in the *C. liquefaciens* PS in comparison with the *C. neoformans* sample probably accounts for these differences.

### Viscosity and elastic properties of PS fractions

**Figure 6:**
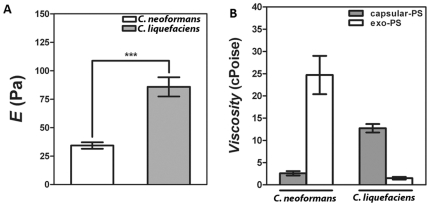
Elastic and viscosity properties of *C. liquefaciens* polysaccharide. Panel A) Young modulus (E) of *C. neoformans* and *C. liquefaciens* capsular-PS demonstrates significant differences in their elastic properties (*** P value<0.01 calculated by *Student's T test (two-tailed)*). Panel B) Viscosity values for *C. neoformans* and *C. liquefaciens* capsular and exo-PS. The differences between exo- and capsular-PS in *C. neoformans* and *C. liquefaciens* were statistically significant (P value<0.01 calculated by *Student's T test (two-tailed)*). Also, the differences between PS types for both yeast species were statistically significant (P value<0.01 calculated by *Student's T test (two-tailed)*).

Young's modulus of capsular-PS of *C. liquefaciens* was determined using an optical tweezers system following the theoretical model previously described [26]. This assessment was simplified since the capsule sizes both yeast species were statistically similar. Young's modulus (E) values of *C. liquefaciens* PS capsule were 86±9 Pa, which were 2.5-fold higher than for *C. neoformans* (Figure 6A, P value<0.01).

The viscosities of *C. neoformans* and *C. liquefaciens* capsular and exo-PS were determined using the diffusion coefficient of beads coated with a PS aqueous solution (1 mg/ml) in an optical tweezers system and following the procedures previously described elsewhere (Figure 6B) [25], [30]. For *C. liquefaciens*, the capsular PS viscosity value was 13±1 cPoise, which was 8-fold higher than *C. liquefaciens* exo-PS viscosity value (1.5±0.3 cPoise; P value<0.01). In contrast, C. *neoformans* capsular-PS viscosity value was 2.6±0.5 cPoise, almost 10-fold lower than *C. neoformans* exo-PS viscosity value (25±4 cPoise; P value<0.01). When both yeast species were compared, capsular-PS viscosity for *C. liquefaciens* was 5-fold higher than for *C. neoformans*. On the other hand, Exo-PS viscosity for *C. liquefaciens* was 16-fold lower than for *C. neoformans*. Differences between similar PS fractions in the two were statistically different (P values<0.01).

### Antigenic and chemical composition of *C. liquefaciens* capsule

**Figure 7:**
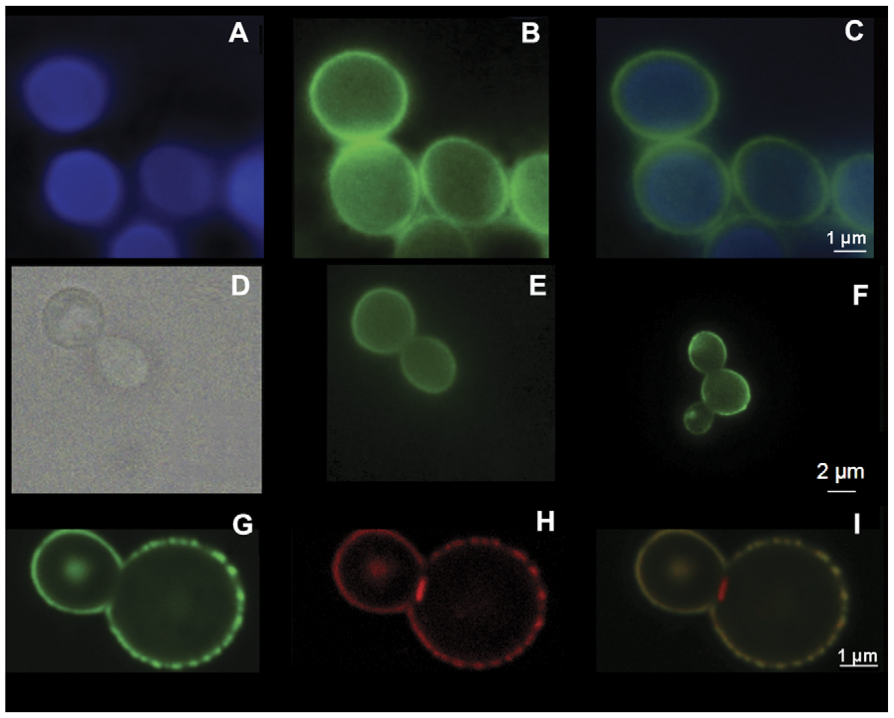
Surface architecture of *C. liquefaciens*. Panel A) Uvitex staining (blue) of *C. liquefaciens* strain showing chitin distribution at the cell wall. Panel B) Staining of *C. liquefaciens* cell with mAb 18B7 showing that the antibody raised to *C. neoformans* GXM cross reacts with *C. liquefaciens* components. Panel C) Merge of panels A and B. Panel D) Light microscopy of acapsular *C. neoformans* cap67 cells. Panel E) Exo-PS from *C. liquefaciens* attaching to an acapsular *C. neoformans* mutant with the PS labeled with mAb 18B7-FITC. Panel F) Incorporation of *C. neoformans* (control) exo-PS by acapsular *C. neoformans* cells with labeling of PS by mAb 18B7-FITC. Panel G) *C. liquefaciens* labeled with 1 mAb 8B7-FITC Panel H) *C. liquefaciens* stained with WGA-rhodamine. Panel I). Merge of G and H with *C. neoformans* labeled with mAb 18B7 (green fluorescence) and WGA (red fluorescence).

Staining of yeast cells with Uvitex revealed that the distribution of cell wall chitin in *C. liquefaciens* was similar to that previously observed for *C. neoformans* [31]–[32] (Figure 7A–C). Since GC-MS analysis revealed the presence of the GXM building units in *C. liquefaciens* samples, we hypothesized that yeast cells or their soluble fractions could be recognized by antibodies raised to *C. neoformans* GXM. These antibodies, in fact, were previously demonstrated to cross-react with different fungi [33]–[34]. Monoclonal antibody (mAb) 18B7 was selected for initial tests, since it reacts with different GXM motifs of all *C. neoformans* and *C. gattii* serotypes [3]. Capsular structures of *C. liquefaciens* were recognized by mAb18B7 (Figure 7B, C, and G), suggesting the presence of common capsular epitopes. The similarities in the capsular domains of *C. neoformans* and *C. liquefaciens* were further confirmed by the fact that a *C. neoformans* acapsular mutant was able to incorporate mAb18B7-reactive components from exo-PS of *C. liquefaciens* onto the cell surface (Figure 7E) in a manner similar to that observed with the incorporation of *C. neoformans* exo-PS onto this acapsular strain (Figure 7F).

The detection of N-acetylglucosamine in DMSO extracts of *C. liquefaciens* capsule by GC-MS led us to evaluate the location of this sugar in the capsule. N-acetylglucosamine has previously been suggested to connect the cell wall to capsular components in *C. neoformans* [31]–[32]. Staining of yeast cells with WGA revealed that β1,4 *N*-acetylglucosamine oligomers are abundant in budding sites, as described for *C. neoformans* (Figure 7G–I). Surprisingly, the lectin also reacted with the entire capsular surface in *C. liquefaciens* yeast cells. In contrast, WGA stains in a localized manner in *C. neoformans*, primarily at regions of budding [32].

### Serological properties of *C. liquefaciens* PS fractions

**Figure 8:**
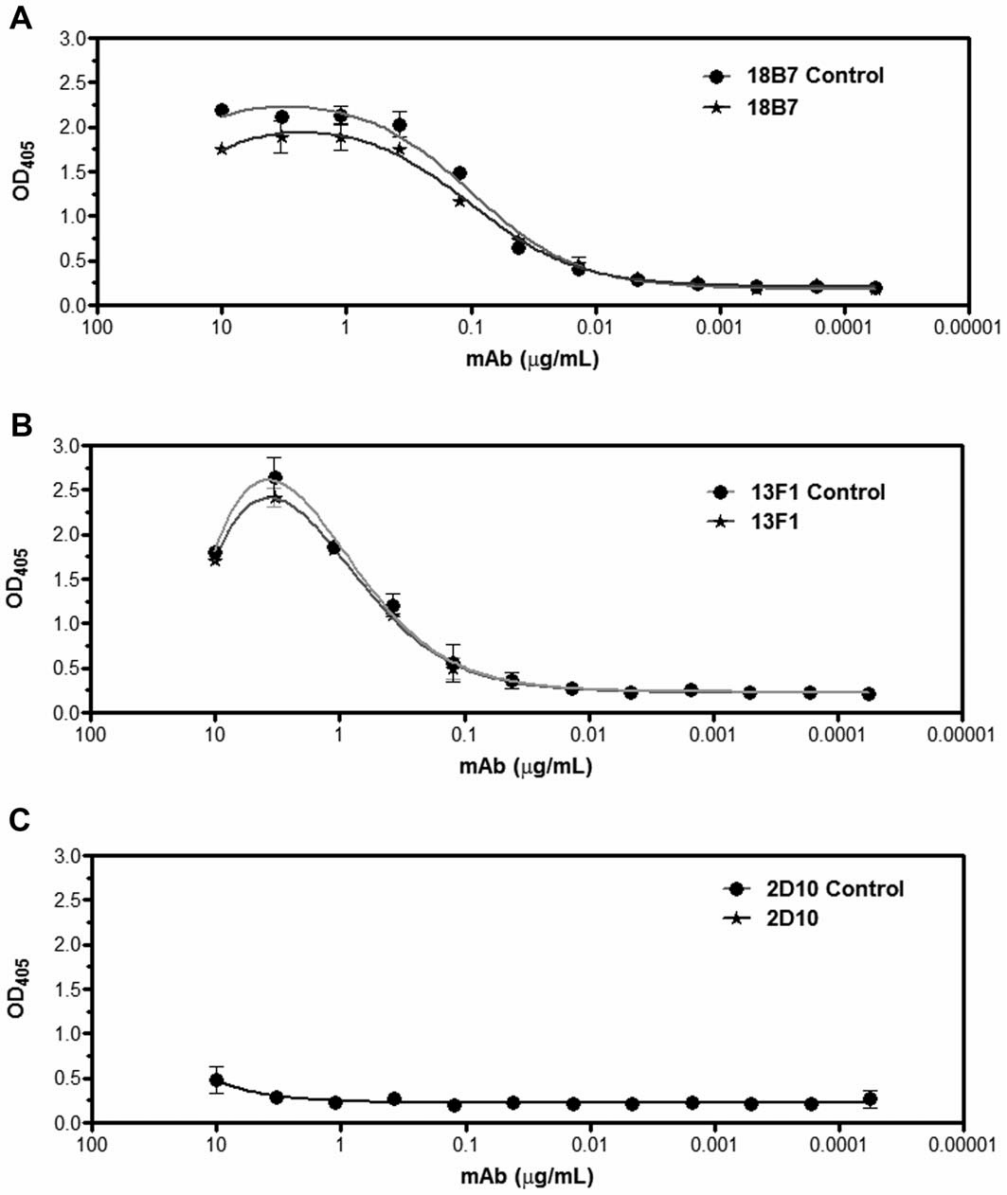
Binding of monoclonal antibodies to *C. liquefaciens* polysaccharide. Serological reactivity of *C. liquefaciens* PS with antibodies 18B7 (panel A; IgG1), 13F1 (panel B; IgM) and 2D10 (panel C; IgM) are shown. *C. neoformans* PS was used in control systems.

To further assess the capacity of *C. liquefaciens* to bind mAbs raised to *C. neoformans* PS, we measured the dissociation constants for PS-mAb reactions with well characterized mAbs to *C. neoformans* GXM by ELISA [35]–[36]. The binding curves of IgG1 18B7, (Figure 8A), IgM 13F1, (Figure 8B) and IgM 2D10 (Figure 8C) were similar for PS from both species. The dissociation constants for the three different mAbs did not differ significantly for *C. liquefaciens* and *C. neoformans* PS samples (Table 2), suggesting that both extracts have comparable antigenic density for the epitopes recognized by these mAbs.

**Table 2:** Dissociation constants (Kd) after binding of mAbs to cryptococcal PS.

PS sample	Kd±Std.error
18B7-Cn^1^	0.099±0.014
18B7-Cl^2^	0.102±0.012
13F1-Cn	1.366±0.217
13F1-Cl	1.239±0.123
2D10-Cn	ND
2D10-Cl	ND

1 Cn: *C. neoformans*.

2 Cl: *C. liquefaciens*.

ND: Not determined.

### Different outcomes after phagocytosis of *C. neoformans* and *C. liquefaciens* by environmental amoebae

**Figure 9:**
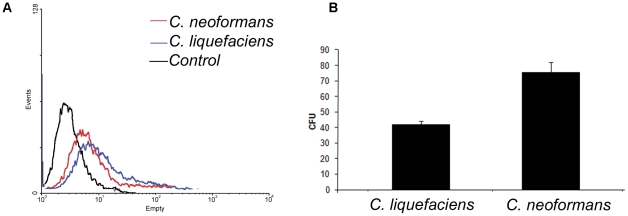
Phagocytosis assays of fungal cells by *Acanthamoeba castellanii*. Panel A) Profiles of interaction of FITC-labeled *C. liquefaciens* at 30°C with *Acanthamoeba castellanii* as determined by flow cytometry. In red, fluorescence population of *C. neoformans* cells in amoebae. In blue, fluorescence population of C. liquefaciens cells in amoebae. In black, non-fluorescence control amoebae. Panel B) Fungal survival (CFU counts) of yeast cells after interaction with amoebae is shown for *C. liquefaciens* and *C. neoformans* at 30°C. All experiments were done in triplicates and *Student's T test (two-tailed)* was carried for statistical studies.

A primary function of *C. neoformans* capsular polysaccharides is protection against phagocytosis and killing by animal and environmental phagocytes [37]–[43]. *C. liquefaciens* and *C. neoformans* exist in environments in which they are likely interacting with predator amoebae. Developing resistance mechanisms to survive predation may facilitate survival within mammalian hosts (e.g. environmental predators serving as a “boot camp” for environmental fungi [3]. Consequently, we evaluated the capacity of *Acanthamoeba castellanii* to phagocytosis and kill *C. neoformans* and *C. liquefaciens*, Yeast cells from both species were phagocytosed at similar rates by amoebae (Figure 9A). However, the survival of intracellular *C. liquefaciens* yeast cells was significantly less compared to *C. neoformans* at 30°C (45% vs 79%. P Value<0.01; Figure 9B). Hence, *C. liquefaciens* was more susceptible to predation by amoeba cells compared to the pathogenic yeast cells.

## Discussion

Many of the virulence factors required for pathogenicity in mammals are also important for fungal survival during interactions with environmental predators (reviewed in [44]). The PS capsule of *C. neoformans* is associated with the capacity of the fungus to cause disease in mammals, but similar structures are also found on environmental *Cryptococcus* species, which are saprophytic or normal flora of small animals. While studying the giant snail *Achatina fulica* microbiota, we recovered several yeast species, including *C. liquefaciens*. *C. liquefaciens* is a member of *Cryptococcus* genus, but there are no reported cases of human disease by this species. Hence, we used *C. liquefaciens* as a non-mammalian pathogenic model to compare and contrast physical-chemical features of the PS from *C. liquefaciens* with PS from *C. neoformans*.

*C. liquefaciens* produced a PS capsule that was similar to the capsule of *C. neoformans* when visualized by light microscopy. *C. liquefaciens* secreted large amounts of PS into the media. Isolated capsular and exo-PS from *C. liquefaciens* contained similar monosaccharide composition when determined by GS-MS as *C. neoformans* PS fractions, but the ratios of the monosaccharides were different in the two species. Glucose was also detected in all fractions, which may reflect the co-extraction of glucans, which are frequently found in DMSO extracts of *C. neoformans* [25]. The amount of glucose was higher in *C. liquefaciens* PS fractions. N-acetylglucosamine was detectedby GS-MS in *C. liquefaciens* capsular and it was also labeled by fluorescent lectin, indicating that this compound is a component of *C. liquefaciens* capsule. Since the two *Cryptococcus* species contained the same monosaccharides, we hypothesized that *C. liquefaciens* cells or their soluble fractions could be recognized by antibodies raised to *C. neoformans* GXM. These antibodies are known to cross-react with different fungi [33], [34]. Similarities in the overall Kd for the mAbs and the PS fractions from both yeast species suggested that the extracts were recognized with comparable affinities by antibodies to GXM. The similarities in the capsular domains of *C. neoformans* and *C. liquefaciens* were further highlighted by the fact that a *C. neoformans* acapsular mutant was able to incorporate mAb18B7-reactive components from *C. liquefaciens* onto the cell surface, suggesting similarities in the mechanisms of capsule anchoring to the cell wall.

Given that the monosaccharide compositions were similar between these two species and that they shared antigenic determinants, we hypothesized that differences in physical parameters could generate differences in interactions with phagocytes. Macro-structural parameters such as, Mw, R_g_ and R_h_ showed significant differences between the species. In particular, the shape factor values provide important knowledge about differences in the degree of branching of PS molecules. The ρ of capsular and exo-PS in *C. neoformans* were values characteristic of linear molecules. However, the ρ of both fractions on *C. liquefaciens* PS were values typical of linear PS [23]. Similarly, the zeta potential, Young modulus and viscosity values of *C. liquefaciens* PS were consistent with linear structures. In contrast, these measurements in *C. neoformans* correspond to PS with branched structures [23].

Studies have established the potential of physical chemical properties of *C. neoformans* and *C. gattii* PS fractions to modulate the host response [23]–[24]. The degree of PS branching can influence the biological properties of *C. neoformans* [23]. Therefore, we hypothesized that *C. liquefaciens'* linear PS capsule would result in different interactions with *A. castellanii* compared to amoebae interacting with the highly branched capsule of *C. neoformans*. Interestingly, both species were phagocytosed at similar rates. However, the intracellular survival of *C. neoformans* was significantly greater than that of *C. liquefaciens*. Although we cannot determine the extent to which the capsule of *C. liquefaciens* contributes to the reduced survival after phagocytosis relative to *C. neoformans*, we can state that the *C. liquefaciens* PS is not sufficient to protect the fungus from predation.

The capsule of *C. neoformans* benefits the fungus during interactions with animal phagocytes and amoeboid predators [40], [41], [44]. The ability of different cryptococcal PS to promote survival during interactions with phagocytes may be a key determinant for the ability of fungal cells to persist and cause disease in mammalian hosts, differentiating opportunistic pathogens from exclusively environmental microbes. Here we demonstrate that *C. liquefaciens*, an environmental yeast, produces a PS capsule that is similar to that produced by the pathogenic members of the *Cryptococcus* genus in many aspects, but the capsular and exo-PS manifest significant structural differences such that they are less efficient in protecting the yeast. Although the virulence barrier for *C. liquefaciens* is almost certainly related to its inability to tolerate mammalian temperatures, our findings reveal that it also lacks melanin and has a less effective, linear capsule. Hence, morphologically similar capsules by light microscopy can have major differences in physical and protective properties.

## Materials and Methods

### Yeast strains

The strain of *C. liquefaciens* used in this study was obtained as described below. *C. neoformans* H99 strain was used as a reference control in all assays. Fungal cells were maintained on Sabouraud solid medium at 30°C. For capsule induction, yeast cells were grown at 30°C in minimal medium (16 mM of glucose, 10 mM of MgSO_4_, 29.4 mM of KH_2_PO_4_ and 13 mM of glycine, pH 5.5). Incubation times depended on the experiment performed. For each experiments, *C. neoformans* and *C. liquefaciens* were growth under the same conditions.

### Isolation of C. liquefaciens

*C. liquefaciens* was isolated from the snail *Achatina fulica*. Briefly, five different animals were captured from the campus of the University of Rio de Janeiro at different periods of time. Approximately 3 ml of digestive juice were collected from the crop and stomach after the animals were anesthetized by immersion into 0.4 mg/mL of sodium pentobarbital for 8 h as described by Zeck-Coelho et al [45]. Samples were then serially diluted in phosphate buffered saline (PBS). A 100 µL volume was plated on Sabouraud agar media and incubated at 30°C for 72 h. Pure cultures were obtained after repeated sub-culturing on Sabouraud agar media. An isolate, named *car17*, was verified as *C. liquefaciens* and selected for use in all subsequent studies.

### Biochemical and molecular identification

#### Growth curves

*Car17* yeast cells were grown in minimal medium for 7 d at 30°C and cell density was measured every 6 h. *C. neoformans* cells were used as controls. Optical density was measured at 540 nm.

#### Urea degradation

*C. liquefaciens* and *C. neoformans* yeast cells were grown for 48 h at 30°C in a differential medium that tests the ability of an organism to produce urease by the hydrolysis of urea to ammonia and carbon dioxide. *Candida albicans* ATCC 10231 was used as negative control.

#### Growth on cycloheximide medium

H99 and *car17* yeast cells were grown in Sabouraud media supplemented with 0.01% of cycloheximide for 5 d at 30°C.

#### Melanization assays

For the *in vitro* melanization assay, H99 and *car17* yeast cells were streaked on chemically defined minimal medium (15 mM glucose, 10 mM MgSO_4_, 29.4 mM KH_2_PO_4_, 13 mM glycine and 3 mM thiamine, pH 5.5), with and without 1 mM L-DOPA (Sigma-Aldrich), and incubated in the dark for 20 days at 30°C. Plates were examined daily to monitor growth and pigment production.

#### Germ tube formation

Production of germ tube formation was tested by incubation of *car16* yeast cells in horse serum at 30 and 37°C for 2 hours.

#### Molecular identification

Genomic DNA was extracted and purified from yeast phase *car17* cells by phenol/chloroform/isoamyl alcohol as described [46]. For partial sequencing, primers UNI1 (5′-GTCAAACTTGGTCATTTA-3′) and ITS4 (5′-TCCTCCGCTTATTGATATGC-3′), targeting the conserved regions of *18 S* and *28 S* rDNAs [47], respectively, were used for amplification. To confirm the result and differentiate between closely related species, the region D1/D2 in 28 S rDNA was analyzed using the primers NL1 (5′-GCATATCAATAAGCGGAGGAAAAG-3′) and NL4 (5′TCCTCCGTCTATTGATATGC-3′). Briefly, we used 5 µl of 10× PCR buffer, 1.5 µl of magnesium chloride (50 mM), 0.4 µl of a deoxynucleoside triphosphate mixture (10 mM each dNTP), 0.8 µl of each primer (40 pmol of each primer), and 0.5 µl (2.5 U) of Platinum®*Taq* DNA Polymerase (Invitrogen, Brazil) in a volume of 50 µl. Amplification consisted of an initial denaturation at 94°C for 4 min; 30 cycles of denaturation at 94°C for 30 s, annealing at 55°C for 30 s, and extension at 72°C for 1 min; and a final extension at 72°C for 4 min; a PCR system thermal cycler (Biorad, USA) was used. Negative control reactions without any template DNA were carried out simultaneously. Automated sequencing was done using the Sequencing Platform at Fundação Oswaldo Cruz - PDTIS/FIOCRUZ, Brazil. Sequences from both strands were generated, edited with the Sequencher ver. 4.6 software package (Genes Codes Corporation, USA), and aligned by means of the Mega version 4.0.2 software. The sequences of our strain were compared by BLAST (Basic Local Alignment Search Tool- NIH) with sequences available from NCBi GenBank.

### Capsule visualization and size measurements by India-ink counter staining

Yeast cells were visualized with an optical microscope (Leica DM5000B, Leica Microsystem, Mannheim, Germany) after suspension in India ink. The capsule size of 100 cells was measured in ImageJ 1.40 g software (http://rsb.info.nih.gov/ij/); National Institutes of Health (NIH), Bethesda, MD).

### Isolation of exopolysaccharides (exo-PS) from culture supernatants by filtration

Based on its ability to self aggregate, PS that accumulated in culture supernatants (exo-PS) were isolated by ultrafiltration using an Amicon (Millipore, Danvers, MA) ultrafiltration cell (cutoff = 100 kDa) as described [30]. The final PS solution was lyophilized and the dry PS mass determined.

### Extraction of capsular polysaccharide (capsular-PS) components with dimethyl sulfoxide (DMSO)

Capsular PS were isolated from yeast cells as described [48]. Briefly, yeast cells were suspended in 15 mL of DMSO and incubated for 30 min at room temperature. DMSO supernatant was collected and then dialyzed extensively against water for 3 d at room temperature. The final PS solutions were lyophilized and the dry PS mass determined.

### Glycosyl composition of PS

PS fractions were dissolved in methanol/1 M HCl and incubated at 80°C for 18 h. Samples were per-*O*-trimethylsilylated by treatment with Tri-Sil (Pierce) for 30 min at 80°C. The per-*O*-TMS derivatives were analyzed by gas chromatography coupled to mass spectrometry (GC-MS) as described [25].

### Labeling of *C. liquefaciens* with probes for the fungal cell surface

Yeast cells (10^6^) were suspended in 4% paraformaldehyde cacodylate buffer (0.1 M, pH 7.2) and incubated for 30 min at room temperature. Fixed yeast cells were washed twice in PBS and incubated in 1% bovine serum albumin in PBS (PBS-BSA) for 1 h at room temperature. The cells were washed again and incubated for 1 h at room temperature in the presence of mAb 18B7 (1 µg/ml). The mAb 18B7 is a mouse IgG1 with high affinity for GXM of different cryptococcal serotypes [3]. After washing in PBS, the cells were incubated with 25 µM Uvitex2B for 1 h at room temperature. Uvitex2B (Polyscience Inc, Warrington, PA) binds to chitin (a polymer of N-Acetyl-D-Glucosamine) in fungal walls. After washing in PBS, the cells were finally incubated with a fluorescein isothiocyanate (FITC) labeled goat anti-mouse IgG (Fc specific) antibody (Sigma) for 1 h at room temperature. In some systems, yeast cells were also stained with the lectin WGA, with known affinity for β1,4 *N*-acetylglucosamine (GlcNAc) oligomers. Cells were suspended in 100 µl of a 5 µg/ml solution of the Alexa Fluor 594 conjugate of WGA (Molecular Probes) and incubated for 30 min at 37°C. Cell suspensions were mounted over glass slides and analyzed with a Leica DM5000B (Leica Microsystem, Mannheim, Germany) fluorescence microscope. Images were processed using ImageJ.

### Polysaccharide binding by acapsular cells

Acapsular *C. neoformans* yeast cells (strain Cap67, 10^6^ cells) were suspended in 100 µl of culture supernatants (4 day cultures) from *C. neoformans* or *C. liquefaciens*. PS in supernatants was normalized to10 µg/ml. The suspension was incubated for 12 h at 25°C and extensively washed with PBS. Control systems consisted of Cap67 cells incubated with sterile medium. For immunofluorescence with mAb 18B7, the cells were fixed with 4% paraformaldehyde and prepared as described above [49].

### Measurement of differential refractive index, molecular mass (Mw) and radius of gyration (R_g_) by Static Light Scattering (SLS)

PS solutions were prepared by solubilizing lyophilized polysaccharide in sterile-filtered, degassed ultra-pure water. All samples were passed through an in-line 0.8 µm syringe filter prior to light scattering analysis to eliminate large aggregates, dust and reduce extraneous sources of refracted or scattered light. Differential refractometry was done using a 620 nm laser source (BI-DNDC; Brookhaven Instruments Corp., Holtsville, NY) to measure the change in refractive index as a function of concentration (*dn/dc*) of the PS samples. Molecular masses and radius of gyration were determined at 25°C by multi-angle laser light-scattering in a molecular weight analyzer (BI-MwA, Brookhaven Instruments Corp., Holtsville, NY) using a 675 nm laser source, as described [25], [50]. The Mw and R_g_ were determined using Berry plots, which is more accurate for large molecular mass polymers.

### Effective diameter and hydrodynamic radius of PS samples analyses by Dynamic Light Scattering (DLS)

The effective diameter and the polydispersity of PS preparations were measured by Quasi-Elastic Light Scattering (QELS) in a 90Plus/BI-MAS Multi Angle Particle Sizing analyzer (Brookhaven Instruments Corp., Holtsville, NY) as described [27]. PS solutions were prepared as described above. Measurements were done at 25°C. The multimodal distributions of particle size diameter were generated by a Non-Negatively constrained Least Squares algorithm (NNLS) based on the intensity of light scattered by each particle. All PS samples were analyzed under the same conditions.

### Zeta Potential measurements

Zeta potential (Z), particle mobility and shift frequency of polysaccharide samples were calculated in a Zeta potential analyzer (ZetaPlus, Brookhaven Instruments Corp., Holtsville, NY) as described [25]. Measurements were done at 25°C.

### Binding of monoclonal antibodies (mAbs) to PS in polystyrene plates

Binding of mAbs to the different PS fractions was evaluated by ELISA [35]. Briefly, polystyrene plates were coated with 1 nM PS samples, calculated from the molar concentrations previously determined by the molecular mass measurements. The plates were blocked with PBS containing 1% of bovine serum albumin. The mAbs to PS 18B7 (IgG1), 2D10 (IgM) or 13F1 (IgM) [35]–[36] were then added to the plates, followed by detection of antibody binding to PS using alkaline phosphatase-labeled secondary antibodies. Colored reactions were developed after the addition of *p*-nitrophenyl phosphate (p-NPP) solutions (Sigma, St. Louis, MO) and quantitative determinations performed by absorbance measurement at 405 nm. All incubations were carried at 37°C for 1 h. Determination of Bmax and Kd was calculated with GraphPad Prism 5.02 by fitting only total binding and assuming that the amount of nonspecific binding is proportional to the concentration of PS. Bmax is the maximum specific binding and Kd is the equilibrium binding Constant meaning the PS concentration needed to achieve a half-maximum binding at equilibrium.

### Measurements of viscosity and elastic properties of *C. liquefaciens* PS using optical tweezers

Young's modulus value of *C. liquefaciens* capsular-PS was determined using Glass-bottom dishes coated with 10 µg/mL of mAb 18B7 and incubated for 1 hour at 37°C. Suspensions of 10^4^ yeast cells in PBS were added to the plates and incubated for 1 h at room temperature. After washing with PBS to remove non-adherent cells, polystyrene beads were added to the plate and the samples were placed in the optical tweezers system. Young's modulus values were determined following the theoretical model previously stated [26]. H99 *C. neoformans* cells were used as controls. Viscosity of Capsular-PS and Exo-PS in aqueous solution at 1 mg/mL solution was determined using an optical tweezers assays as previously described [30], [51]. *C. neoformans* capsular-PS and exo-PS samples were similarly measured.

### Phagocytosis assays in amoeba

For interaction of yeast cells with *A. castellanii*, amoeba cells were grown in peptone yeast extract glucose broth (PYG; ATCC medium 354). *A. castellanii* cells were plated in 24-well plates at the density of 10^6^ cells/ml and incubated 1 h at 28°C for adherence to the plastic surface. Suspensions of FITC-labeled *C. neoformans* and *C. liquefaciens* cells were then added at a density of 1×10^6^ cells/ml and the co-cultures were incubated for 1 h at 28°C. Infected amoebae were removed from the plates by scraping for flow cytometry analysis on a FACSCalibur (BD 298 Biosciences, San Jose, CA) flow cytometer. Data were processed with CellQuest (BD Biosciences) and WinMDI (Salk Flow Cytometry). The index of infection was determined as the percentage of fluorescent to non-fluorescent amoeba cells. To discriminate adhered and internalized fungi, infected amoebas were treated for 10 min at 25°C with Trypan blue (200 µg/ml), which quenches the fluorescence of external FITC-labeled fungal cells. Control preparations consisted of amoebae that were not exposed to yeast cells or of amoebae that were infected with unstained yeast (data not shown).

For determination of fungal killing after interaction with *A. castellanii*, amoeba cells were distributed in 96-well plates (10^5^ cells per well) and interactions between these amoebae and fungal cells were analyzed using the conditions described above. Amoebae were then lysed with sterile cold water and the resulting suspension plated onto Sabouraud agar plates. After 48 h of cultivation at room temperature, the number of colony-forming units (CFU) was determined. All experiments were done in triplicates and *Student's T test (two-tailed)* was carried for statistical studies.

### Statistical analysis

Statistical analyses were carried in Bi-ZPMwA Zimm Plot Software (Brookhaven Instruments Corp., Holtsville, NY) for *Mw*, *R_g_* and *A_2_*. 90Plus/BI-MAS Software was used for effective diameter, polydispersity and diffusion coefficient parameters (Brookhaven Instruments Corp., Holtsville, NY). Zeta Plus software was used for Zeta Potential, Mobility and Frequency Shift data (Brookhaven Instruments Corp., Holtsville, NY). Plots, curve fits, and statistical analysis were performed using GraphPad Prism version 5.0a, GraphPad Software, San Diego California USA.
